# The draft mitochondrial genome of *Magnolia biondii* and mitochondrial phylogenomics of angiosperms

**DOI:** 10.1371/journal.pone.0231020

**Published:** 2020-04-15

**Authors:** Shanshan Dong, Lu Chen, Yang Liu, Yaling Wang, Suzhou Zhang, Leilei Yang, Xiaoan Lang, Shouzhou Zhang

**Affiliations:** 1 Fairy Lake Botanical Garden, Shenzhen & Chinese Academy of Sciences, Shenzhen, China; 2 Xi’an Botanical Garden, Xian, China; University of Western Sydney, AUSTRALIA

## Abstract

The mitochondrial genomes of flowering plants are well known for their large size, variable coding-gene set and fluid genome structure. The available mitochondrial genomes of the early angiosperms show extreme genetic diversity in genome size, structure, and sequences, such as rampant HGTs in *Amborella* mt genome, numerous repeated sequences in *Nymphaea* mt genome, and conserved gene evolution in *Liriodendron* mt genome. However, currently available early angiosperm mt genomes are still limited, hampering us from obtaining an overall picture of the mitogenomic evolution in angiosperms. Here we sequenced and assembled the draft mitochondrial genome of *Magnolia biondii* Pamp. from Magnoliaceae (magnoliids) using Oxford Nanopore sequencing technology. We recovered a single linear mitochondrial contig of 967,100 bp with an average read coverage of 122 × and a GC content of 46.6%. This draft mitochondrial genome contains a rich 64-gene set, similar to those of *Liriodendron* and *Nymphaea*, including 41 protein-coding genes, 20 tRNAs, and 3 rRNAs. Twenty cis-spliced and five trans-spliced introns break ten protein-coding genes in the *Magnolia* mt genome. Repeated sequences account for 27% of the draft genome, with 17 out of the 1,145 repeats showing recombination evidence. Although partially assembled, the approximately 1-Mb mt genome of *Magnolia* is still among the largest in angiosperms, which is possibly due to the expansion of repeated sequences, retention of ancestral mtDNAs, and the incorporation of nuclear genome sequences. Mitochondrial phylogenomic analysis of the concatenated datasets of 38 conserved protein-coding genes from 91 representatives of angiosperm species supports the sister relationship of magnoliids with monocots and eudicots, which is congruent with plastid evidence.

## Introduction

Plant mitochondrial (mt) genomes are about 100–10,000 times larger than those of animals and are structurally more complex due to frequent ongoing recombinations [1]. The notably large size of plant mt genomes is shaped by a combination of several factors, including a rich gene set along with the abundant introns that carries, and the capability of uptaking and integrating intracellular transferred sequences from the chloroplast [2] and nucleus [3], and horizontally transferred genes from foreign donors [4, 5]. Based on the database of the available plant mt genomes (https://www.ncbi.nlm.nih.gov/genome/organelle/), species from each of the three bryophyte lineage hold rather stable mt genome size, conserved gene content, and similar gene order [6–8], whereas the mt genomes of vascular plants demonstrate significant genome size variation, gene set variability, and structural dynamics [9–11]. In particular, vascular plant mt genomes range in size from 66 Kb [12] to 11.3 Mb [13] with encoded genes from 13 to 64 [14]. Neither of the two vascular plant mt genomes sequenced to date, even those of different accessions from the same species, share the same gene order [8], which is in stark contrast to the conserved structural evolution of the plastid genomes of the land plants [15].

The structural fluidity of vascular plant mt genomes is associated with the recombination activity of the repeated sequences [9, 16], such as, it has been proposed that intragenomic homologous recombination via inverted repeats would lead to an inversion, and direct repeats lead to a subdivision of the main genome into sub-circles [17]. As a result, vascular plant mt genome generally contains, in coexistence of the master circle conformation, a variety of rearranged molecules (alternative conformation) in substoichiometric levels. If one of those structural variants is passed on to the progeny, then the gene order might be changed within two generations [18], as observed in a few species with large DNA insert libraries [3, 9, 13, 18–25] or/and third-generation sequencing reads [26, 27]. These studies also suggested repeat recombination frequency to be associated with the repeat length and identity. Generally, large repeats (>1000 bp) with high sequence similarity tend to recombine more frequently, medium repeats (100–1000 bp) recombine occasionally, and small repeats (<100 bp) rarely. Some studies have also suggested the adaptive value of repeat recombination against desiccation in vascular plant mitochondrion [28]. However, it remains a big challenge to predict the specific repeats recombining and to study the functional consequences of the mitochondrial repeat recombination, due to limited data.

Angiosperms, with nearly 250,000 species, represent the most diverse of all major lineages of land plants and the dominant vegetation in earth’s terrestrial ecosystems [29]. It would be of considerable interest to understand the genome evolution of the nuclear, plastid and mitochondrial of angiosperms, especially those of the early diverging lineages. The Four available mt genomes of the early angiosperms show extreme diversities on many aspects: The enormous, 3.9-Mb mt genome of *Amborella trichopoda* contains six genome equivalents of foreign mtDNAs, acquired from green algae, mosses, and other angiosperms [5]. The 617-Kb mt genome of *Nymphaea colorata* holds the most abundant repeats (~50% of genome, 83,705 repeats) among that of the land plants whereas only a few of these show recombinational evidence [27]. The 1.1-Mb mt genome of *Schisandra sphenanthera* (NC_042758, unpublished) holds huge portions of promiscuous sequences (656 Kb, 60%), but small amount of repeats (49 Kb, 4%). The 553-Kb mt genome of *Liriodendron tulipifera* is conserved in gene content and gene order with extraordinarily low mutation rate [30]. Expanded sampling of the mt genomes of early angiosperms would allow more insights into the mitogenomic diversity and evolution of angiosperms.

As a phylogenetically early assemblage of angiosperms, magnoliids contain remnants of many of the oldest lineages of angiosperms and occupy a pivotal position in the phylogeny of angiosperms. Recently, two independent phylogenomic analyses including each of the two newly reported nuclear genomes of magnoliids have led to controversial taxonomic placements of magnoliids [31–33]. Specifically, magnoliids (with *Cinnamomum kanehirae* as the only representative) is resolved as the sister to eudicots with relatively strong support [32], which is consistent with the result of the phylotranscriptomic analysis of the 1–kp data [34] and of 20 representative transcriptomes [35]. Alternatively, magnoliids (with *Liriodendron* as the only representative) is resolved as the sister to eudicots and monocots with weak support [33], which is congruent with the plastome phylogenomic analysis of land plants and Viridiplantae [36, 37]. The controversial taxonomic placements of magnoliids relative to monocots and eudicots between plastid and nuclear evidence need to be further tested with mitochondrial phylogenomic analyses. The slow-evolving, uniparentally-inherited, non-recombining mitochondrial genome sequences are less suffered from the effects of substitution saturation, incomplete lineage sorting, and hybridization commonly seen in nuclear markers, therefore are more suitable for phylogenetic inferences of higher taxonomic categories [38]. In addition to sequence level, plant mt genomes can also provide phylogenetic information on the structure level. The accumulation of the mt genomes of angiosperms, especially those of early diverging lineages would provide us a good opportunity to examine the phylogenetic position of magnoliids using mitochondrial phylogenomic analyses.

*Magnolia biondii* Pamp. (Magnoliaceae, magnoliids) is a deciduous tree species widely grown and cultivated in the north-temperate regions of China for its ornamental and pharmaceutical values. The dried flower buds of *M*. *biondii* (herbal name, Xin-Yi) are a traditional Chinese medicine with a long history of clinical use in the treatment of allergic rhinitis and sinusitis [39]. Modern phytochemical studies have characterized the chemical constitutes of the volatile oil [40], lignans [41], and alkaloids [42] from different parts of the plant *M*. *biondii*, whereas the genetic background of this species is still understudied with only the plastid genome (KY085894, Unpublished) deposited in the GenBank. Here we sequenced and assembled the draft mt genome of *M*. *biondii* using the Oxford Nanopore sequencing technology to study the mitogenomic diversity and the evolution of the early flowering plants.

## Materials and methods

### Mitochondrial genome assembly and annotation

The mt genome of *M*. *biondii* was obtained from the genome project of *M*. *biondii* led by Shouzhou Zhang (unpublished data). The total genomic DNA of *M*. *biondii* was extracted using a modified CTAB method [43] and quality controlled using Agarose gel electrophoresis and Nanodrop 2000 Spectrophotometer (Thermo Fisher Scientific, USA). Single molecule sequencing of the *Magnolia* genomic DNA was performed on the Oxford Nanopore PromethION sequencing platform in Nextomics (Nextomics Biosciences Co., Ltd., Wuhan, China). The raw reads in fastq format were corrected, trimmed, and *de novo* assembled using Canu [44]. One mt contig of 967,100 bp, with an average read coverage of 122 × (SRR9720304, S1A Fig), was retrieved from the genome assembly results with Blastn using the 41 protein-coding genes of *Liriodendron tulipifera* (KC821969) as the reference. This mt contig was further polished with 10X genomics reads (S1 Table) generated by BGI-SEQ500 (BGI, Shenzhen) using software Pilon [45] for three rounds of error correction. The resultant mt contig was elongated in both ends with Canu corrected long reads using BWA [46], yielding a circular molecule of 995,279 bp (S1B Fig). We mapped all the corrected genome reads to the circular molecule, but observed uneven read coverage of this putative mt genome. The newly elongated region received very low coverage (~7 ×) in the reads mapping file (SRR9720674, S1B Fig). Therefore, to be cautious, our subsequent analyses were based on the corrected mt contig of 967,100 bp.

**Fig 1:**
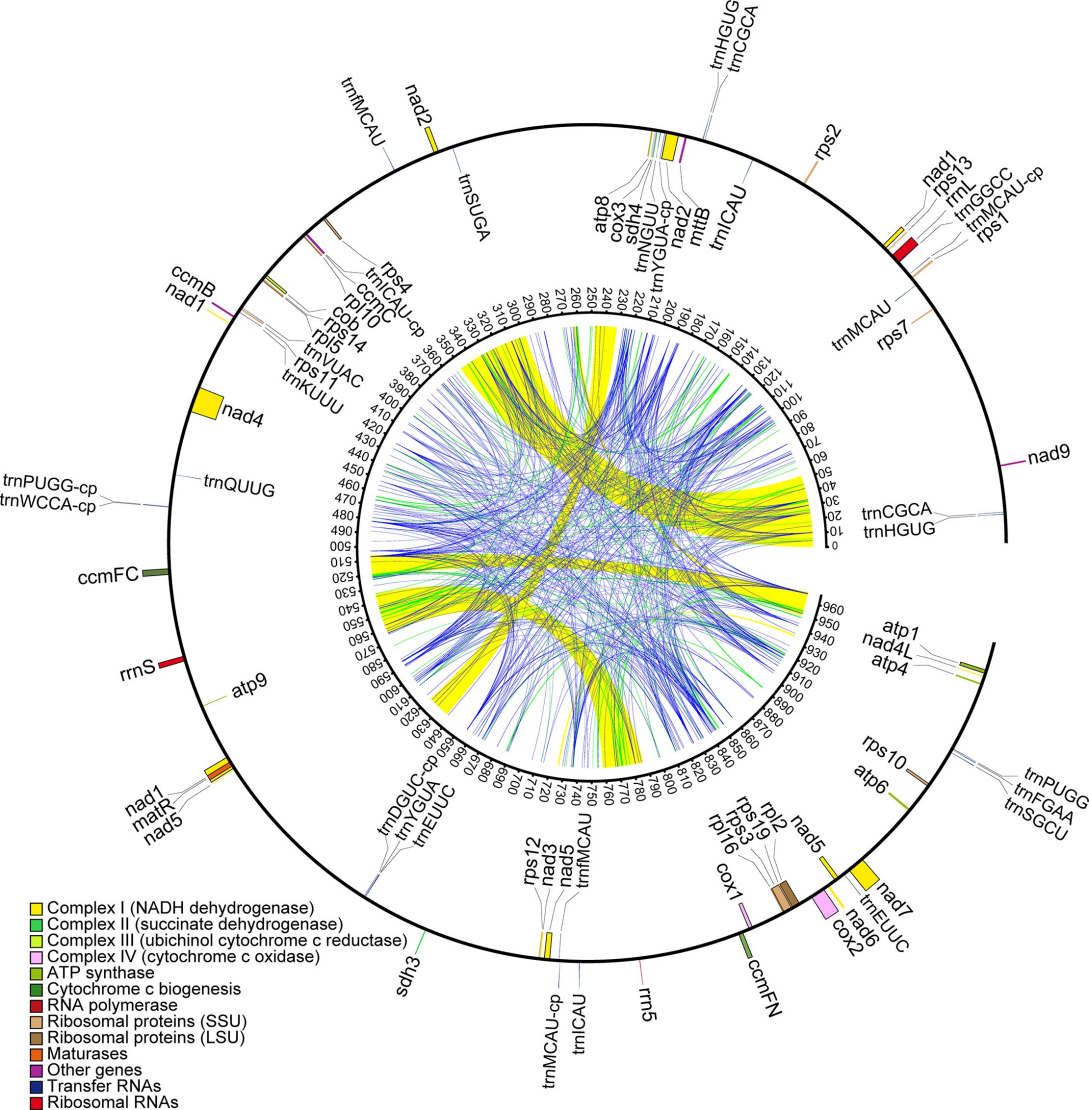
Draft mitochondrial genome map of *Magnolia biondii*. The total length of the *Magnolia* draft mt genome is 967,100 bp. Genes (exons are shown as closed boxes) shown outside the curve are transcribed clockwise, whereas those inside are transcribed counter-clockwise. Genes from the same protein complex are colored the same, introns are indicated in white boxes, and tRNAs of chloroplast origin are noted with a ‘-cp’ suffix. Repeat distributions and occurrences are show inside the gene map. Large repeats >1,000 bp in length are indicated in yellow, medium-sized repeats in the range of 100–1,000 bp in length are indicated in green, and small repeats <100bp in length are colored blue. Numbers on the inner curve represent genome coordinates (Kb).

The annotation for the draft mt genome of *Magnolia* was performed as previously described [6, 47]. Protein coding genes and rRNA genes were annotated by Blastn searches of the non-redundant database at National Center for Biotechnology Information (NCBI). The exact gene and exon/intron boundaries were confirmed in Geneious software (v.10.0.2, Biomatters, www.geneious.com) by mapping the RNA-seq reads (S1 Table) to the mt genome of *Magnolia* using Bowtie2 [48] and further validated by aligning each gene to its orthologs from available annotated plant mitochondrial genomes at the NCBI website (www.ncbi.nlm.nih.gov/genome/organelle). The tRNA genes were detected using tRNAscan-SE 2.0 [49]. Nuclear and plastid homologous sequences were annotated by searching the *Magnolia* mt genome against the nuclear (unpublished) and the chloroplast genome of *M*. *biondii* (KY085894, unpublished) using Blastn with an e-value cut-off of 1e^-6^. The mtDNA sharing of *Magnolia* with the mt genomes of other angiosperms was also performed with Blastn with the same parameters using the intergenic spacer regions of *Magnolia* mt genome as the query. The annotated *Magnolia* mt genome was submitted to GenBank under the accession number of MN206019 and visualized using OGDraw 1.2 [50] to generate the genome map (Fig 1).

### Repeats and repeat-mediated homologous recombinations

Repeat identification of *Magnolia* and other angiosperm plant mt genomes was carried out using the python tools as described by Wynn & Christensen [28]. Repeats were counted in three categories, large repeats above 1000 bp, medium repeats in the range of 100–1000 bp, and small repeats between 50 and 100 bp. For the detection of the ongoing repeat-mediated intragenomic recombinations, we set up a mt read database from all the corrected Nanopore reads. We used the *Magnolia* mt genome sequence as the reference to blast the total genomic read database with an e-value cut-off of 1e^-6^ for the extraction of mt reads. Finally, we got a mt read database of 174,003 reads with an average length of 22,527 bp, and a total length of 3,919,721,246 bp.

Repeat-mediated homologous recombinations were evaluated for those repeat pairs ranging from 50 to 29,306 bp with blast identity > 85% following Dong et al. [27]. Specifically, for each repeat pair, we built four or eight reference sequences, each with 1000 bp up- and down-stream of the two template sequences (original sequences), and two (for repeat pair with identity equals100%) or six (for repeat pair with identity less than100%) recombinant sequences (alternative conformations) constructed from the putative recombination products, respectively (S3 Fig). Then, we searched these recombinant sequences against the *Magnolia* mt genome sequence to remove those located in the genome. After that, we blasted the remaining reference sequences against the *Magnolia* mt reads database, and count the number of matching reads with blast identity > 95%, and a hit coverage of at least 200 bp in both flanking regions of each repeat sequence.

### Phylogenetic analysis

For mitochondrial phylogenomic analyses of angiosperms, we downloaded 82 representative mt genomes of vascular plants from the NCBI Organelle Genome Resources database (http://www.ncbi.nlm.nih.gov/genome/organelle/), including two gymnosperm outgroups and 81 angiosperm ingroups with only one representative per genus. These representative mt genomes were selected based on the quality of annotation and the number of encoded-genes. These mt genomes comprise 24 angiosperm orders with an emphasis on eudicots (16 orders) and monocots (4 orders). The early angiosperms were represented by only five species: *Liriodendron tulipifera*, *Magnolia biondii*, *Schisandra sphenanthera*, *Nymphaea colorata*, and *Amborella trichopoda*. To have a good representation of magnoliids and early angiosperms, we downloaded 10 SRA accessions of whole genome sequencing sequences from magnoliids (5), Austrobaileyales (2), Nymphaeales (2), and Chloranthaceae (1). Overall, the mitochondrial phylogenomic analyses in our study comprised 91 representatives of angiosperms, which represented 91 genera, 43 families, 28 orders of APG IV [51]. Our sampling covers all the three so-called ANA grade (Amborelllales, Nymphaeales, Austrobaileyales) [52], and all the five mesangiosperms (*Ceratophyllum*, Chloranthales, magnoliids, eudicots, and monocots) lineages but *Ceratophyllum* for which the available sequencing data is from the targeted sequencing of the nuclear genes and yielded no mt genes for our study. We included 7 representatives from magnoliids, covering all the four orders, Canellales (representated by *Warburgia ugandensis*), Laurales (representated by *Cinnamomum micranthum f*. *kanehirae* and *Persea americana*), Magnoliales (representated by *Magnolia biondii* and *Liriodendron tulipifera*), and Piperales (representated by *Peperomia macraeana* and *Piper auritum*). This taxonomic sampling scheme was designed to reconstruct an overall angiosperm phylogeny, and to infer the phylogenetic relationship of magnoliids relative to monocots and eudicots.

For the downloaded mt genomes, we extracted 38 conserved mitochondrial protein-coding genes in Geneious 10.0.0 (www.geneious.com) for subsequent phylogenetic analysis, including, *atp1*, *atp4*, *atp6*, *atp8*, *atp9*, *ccmB*, *ccmC*, *ccmFC*, *ccmFN*, *cob*, *cox1*, *cox2*, *cox3*, *matR*, *mttB*, *nad1*, *nad2*, *nad3*, *nad4*, *nad4L*, *nad5*, *nad6*, *nad7*, *nad9*, *rpl10*, *rpl16*, *rpl2*, *rpl5*, *rps1*, *rps3*, *rps4*, *rps7*, *rps10*, *rps12*, *rps13*, *rps14*, *rps19*, and *sdh4*. For the SRA sequencing reads, we extract the conserved mt genes using bioinformatics pipeline Hybpiper [53] with the protein sequences of 38 conserved mt genes from 40 representative angiosperms as the bait references. All the gene matrices were parsed with custom Perl script to remove those harboring premature stop codons and blasted against NCBI nucleotide database to remove potentially HGTs. All the genes were firstly evaluated for substitution saturation using DAMBE5 [54] for three codon positions, respectively. As substitution saturation was not detected for any of these mt genes, we included all the mt genes in our phylogenetic analyses.

Each mitochondrial gene was aligned using a local version of TranslatorX [55]. The program first translates the nucleotide sequence into an amino acids sequence using the standard genetic code, and then uses MAFFT [56] to create an amino acid alignment. The alignment is further trimmed for ambiguous portions by GBLOCKS [57] with the least stringent settings. The cleaned amino acid alignment is then used as a guide to generate the nucleotide sequence alignment. The resulted individual mitochondrial alignments were concatenated into combined datasets using the software FASconCAT-G [58]. The concatenated datasets for amino acids (AA) and nucleotides (NT) were analyzed using Partitionfinder [59] for best-fit models and partition schemes, and RAxML v7.2.3 [60] for subsequent phylogenetic tree reconstruction with the maximum likelihood (ML) method with 500 bootstrap replicates. Bayesian inferences were performed in MrBayes [61] using the same partition schemes and best-fit models as estimated by Partitionfinder [59]. In both cases, two independent analyses were run for a total of 10,000,000 generations of Monte Carlo Markov chains and a sampling frequency of 1000 generation. After discarding the first 25% of the trees as burn-in, maximum credibility trees were constructed using TreeAnnnotator v.1.7.5 [62], visualized and rooted in Figtree v1.4.1 [63].

## Results and discussion

### Genome sequencing and assembly

Nanopore sequencing of the total genomic DNA produced 12,836,970 reads with an average read length of 13,492 bp (S1 Table). After the correction step, we got 5,858,689 reads with an average length of 14,839 bp. The corrected reads were trimmed and *de novo* assembled in Canu [44]. After that, we retrieved from the genome assembly a single linear mt contig of 967,100 bp with an average read coverage of 122 × (S1A Fig). This mt contig was further corrected using paired-end reads generated by BGI-SEQ500 (BGI, Shenzhen) using the software Pilon [45]. There are several large repeats in the corrected mt contig, including one direct repeat of 52 Kb (in the position of 1–52,072 bp and 298,360–350,756 bp), which is too long even for Nanopore reads to bridge across. The extension of the left side of the mt contig would lead to the duplication of the region between positions 52,073 and 298,359 bp (S2A Fig). Therefore, the mt contig was elongated only in right end and the extended sequence revealed overlaps with the region of 95,120–155,401 bp, yielding a circular molecule of 995,279 bp (S1 Fig). However, the elongated region received very low coverage (~7 ×) in the whole genome read mapping file (S1B Fig), indicating that this putative circular molecule might be an alternative conformation in substoichiometric level. Although *in vivo* existences of the linear or/and branched mt genomes were proposed [64] and could also take place in *Magnolia* mitochondrion, we prudently decided to refer to the original mt contig as the draft mt genome of *Magnolia*.

### Genome size and gene content

The draft mt genome of *Magnolia* is a linear molecule of 967,100 bp with a GC ratio of 46.6% (Genbank accession: MN206019; Fig 1). This is nearly twice the size of *Liriodendron* with a genome size of 554 Kb (Table 1). The relatively large genome size of *Magnolia* is associated with the expansion of the intergenic spacers that reached 890 Kb, accounting for 92% of the genome size. The amount and the proportion of the intergenic spacers of *Magnolia* are notably larger than that of the *Nymphaea* (519 Kb, 84%) and *Liriodendron* (479 Kb, 85%). The intergenic spacer regions are usually packed with repeated sequences, nuclear and plastid transferred sequences, horizontally transferred sequences, and promiscuous sequences of unknown origin. The *Magnolia* mitogenomic spacers contain a total of 1,145 identified repeat sequences, accumulating to 262 Kb (30% of the spacers), which is slightly less than that of the highly repetitive *Nymphaea*, but two times larger than that of the *Liriodendron* and four times larger than that of *Schisandra*. The nuclear and plastid homologous sequences of *Magnolia* mitogenomic spacers add up to 288 Kb (32% of the spacer regions), and 26 Kb (3% of spacer regions), respectively. In contrast to the relatively large amount of nuclear homologous sequences in other three early angiosperms, *Magnolia* nuclear genome sequence transfers might not play such a significant role in the spacer expansion of its mt genome.

**Table 1:** General features of the mitochondrial genomes of the five early angiosperms.

Genome feature	*Amborella trichopoda*	*Nymphaea colorata*	*Schisandra sphenanthera*	*Liriodendron tulipifera*	*Magnolia biondii*
Accession	KF754799–KF754803	KY889142	NC_042758	KC821969	MN206019
Size (bp)	3,866,039	617,195	1,101,768	553,721	967,100
GC%	45.90%	45.10%	46.40%	47.70%	46.60%
Genes	63	64	64	64	64
tRNAs	20	20		20	20
rRNAs	3	3	3	3	3
Protein coding genes	40	41	41	41	41
Cis-spliced introns	19	19	19	20	20
Trans-spliced introns	6	6	6	5	5
Gross length of repeats (Kb)	914 (24%)	302 (49%)	49 (4%)	86 (16%)	264 (27%)
Plastid-derived (Kb)	138 (4%)	13 (4%)	43 (4%)	25 (4%)	26 (3%)
Nuclear-homologous sequences of the spacers (Kb)	1,486 (38%)	429 (70%)	—	427 (77%)	289 (30%)
Total gene length (Kb)	78 (2%)	98 (16%)	78 (7%)	75 (14%)	77 (8%)
Protein exons (Kb)	34 (1%)	36 (6%)	36 (3%)	35 (6%)	35 (4%)
Cis-spliced intron length (Kb)	39 (1%)	55 (9%)	33 (3%)	34 (6%)	37 (4%)

*Magnolia* shares 80% (595 Kb) of its spacer region with the other sequenced plant mt genomes. Among early angiosperms, *Magnolia* mt genome shares its intergenic spacers the most with that of the *Liriodendron* (358 Kb), followed by *Amborella* (203 Kb), *Schisandra* (148 Kb), and finally, *Nymphaea* (45 Kb). The high mtDNA sharing level between *Magnolia* and *Liriodendron* might reflect their relatively recent divergence time (ca. 55 Mya, www.timetree.org) and lower sequence turnover rate [23]. Angiosperm mt genomes are highly divergent because rapid structural evolution induced by recombinations could frequently result in losses of gene synteny as well as the mtDNA sequence fragments [19]. For example, in Fabaceae, the average amount of mtDNA sharing among species with a divergence time of 50 Mya is ca. 170 Kb [65], which is only half that between *Magnolia* and *Liriodendron*.

The *Magnolia* mt genome encodes 64 unique genes, including 41 protein coding genes, 20 tRNAs (14 mitochondrial native and 6 plastid derived), and 3 rRNAs (*rrn5*, *rrn18*, and *rrn26*) (Table 1). Total gene length adds up to 8% of the total mt genome length, with protein-coding regions comprising only 4% (35 Kb) of the genome length. In general, the gene content of *Magnolia* is very similar to the other published mt genomes of early angiosperms, especially to *Liriodendron* [66], *Schisandra* (NC_042758) and *Nymphaea* [27]. The *Magnolia* mt genome contains 25 group II introns disrupting 10 genes, including 20 cis-spliced and five trans-spliced introns (*nad1i394g2*, *nad1i669g2*, *nad2i542g2*, *nad5i1455g2*, *nad5i1477g2*), which is identical to the intron set of *Liriodendron*, but differs from *Schisandra*, *Nymphaea* and *Amborella* by its cis-spliced *nad1i728g2*, which is a trans-spliced intron in the latter three. Overall, the draft genome of *Magnolia* retains a similarly rich gene and intron set as that of the available four early angiosperms, suggesting that the mt genomes of the last common ancestor of flowering plants might possess a rich 41 protein-coding gene set with 25 group II introns, 20 tRNAs, and 3 rRNAs, which is followed by subsequent lineage specific losses of genes and introns in different lineages, alternatively, these earliest angiosperms might have independently gone through similar processes in gene losses and gains in the mt genome evolution.

### Repeats and recombination rate

The draft mt genome of *Magnolia* contains 1,145 repeated sequences that are longer than 50 bp, accounting for 27% of the genome. The repeated sequences in *Magnolia* mt genome contain large proportions (54%) of the medium and large repeats, suggesting potentially more frequent recombinations in the *Magnolia* mitochondrion. We have checked all these repeats for recombination evidences with our long reads database. Surprisingly, no evidence of recombination is detected other than the 17 repeats shown in Table 2. The recombination equilibrium is detected in three largest repeats, including two inverted repeats of 16 Kb and 3 Kb and one direct repeats of 29 Kb. Longer repeat sequences show higher recombination rate, and inverted repeats are more prone to recombination than direct repeats.

**Table 2:** Recombination frequency of the 17 recombinationally active repeats in the mitochondrial genome of *Magnolia biondii*.

Repeat no.	Repeat length	Identity	Start	End	Start	End	Direction	Reads supporting alternative conformation	Reads supporting master conformation
1	29,306	99.56	757,889	787,141	533,954	563,196	Direct	8 (47.06%)	9 (52.94%)
2	15,874	99.45	620,421	636,259	231,809	247,637	Inverted	27 (49.09%)	28 (50.91%)
3	2,822	99.33	949,369	952,184	259,868	262,681	Inverted	86 (51.50%)	81 (48.50%)
4	1,029	98.74	933,397	934,421	725,278	726,297	Inverted	19 (7.98%)	219 (92.02%)
5	746	99.46	520,696	521,439	259,868	260,611	Direct	2 (0.74%)	269 (99.26%)
6	651	99.23	121,635	122,282	94,466	95,114	Inverted	8 (3.05%)	254 (96.95%)
7	495	99.39	947,714	948,208	919,248	919,739	Inverted	4 (1.76%)	223 (98.24%)
8	463	99.14	465,328	465,787	325,427	325,888	Inverted	1 (0.35%)	284 (99.65%)
9	462	99.13	465,328	465,787	26,800	27,259	Inverted	1 (0.35%)	283 (99.65%)
10	268	100	736,243	736,510	545,526	545,793	Direct	3 (0.93%)	319 (99.07%)
11	268	99.25	769,472	769,737	736,243	736,510	Direct	3 (0.94%)	318 (99.06%)
12	180	95	950,890	951,065	543,270	543,448	Inverted	1 (0.25%)	396 (99.75%)
13	162	97.53	950,906	951,065	767,221	767,382	Inverted	1 (0.26%)	392 (99.74%)
14	160	100	543,270	543,429	260,987	261,146	Direct	1 (0.25%)	400 (99.75%)
15	154	100	521,585	521,738	521,534	521,687	Direct	1 (0.46%)	219 (99.54%)
16	103	100	604,952	605,054	597,457	597,559	Direct	1 (0.38%)	260 (99.62%)
17	103	100	521,636	521,738	521,534	521,636	Direct	1 (0.45%)	221 (99.55%)

The length of the repeats and recombination rate are clearly correlated, with no recombination evidence detected for repeat sequences of shorter than 100 bp. Recombination between direct repeats in a master-circle conformation of mtDNA could produce two sub-circles, while recombination between inverted repeats, an inversion. With recombination between these and other repeats, *Magnolia* mtDNAs contain predominant existence of master conformation along with many other alternative conformations with inversions and/or subcircles. This mtDNA heteroplasmy may potentially provide more genetic materials for evolutionary selection [67], hence conferring on *Magnolia* some ecological and genetic fitness during its evolution.

### Plastid derived mitogenomic sequences

*Magnolia* mt genome contains 54 plastid insertions from 54 bp to 4 kb (Table 3) with the total length adding up to 26 Kb, comprising 3% of the genome, which is rather uniform in angiosperm mt genome in terms of both quantity and ratio. The transfer of plastid DNA to the mitochondrion most likely occurred in the ancestor of vascular plants [27]. These plastid transferred DNAs sometimes carries plastid genes, and the transferred protein-coding genes usually became nonfunctional, whereas the tRNA genes mostly remain functional. In *Magnolia* mt genome, we annotated six plastid derived tRNAs, including *trnDGUC*-cp, *trnMCAU*-cp, *trnNGUU*-cp, *trnICAU*-cp, *trnPUGG*-cp, and *trnWCCA*-cp. The transfer of these tRNAs could be dated back to different evolutionary stages of vascular plants. For example, the transfers of *trnHGUG*-cp and *trnMCAU*-cp might have happened in the common ancestor of the seed plants with their earliest occurrence in some gymnosperms [30]. The plastid-derived *trnDGUC*-cp mostly occurs in the mt genomes of some dicots but not in monocots and gymnosperms, therefore the presence of this tRNA in *Magnolia* and *Liriodendron* might represent their earliest emergence in time [30]. This suggests either paralleled gains of this tRNA once in Magnoliaceae and then once again in the ancestor of dicots, or as a single-gain event before the split of magnoliids from the rest of angiosperms, followed by subsequent lineage specific losses in monocots.

**Table 3:** Plastid insertions in the mitochondrial genome of *Magnolia biondii*.

Plastid insertion	Minimum	Maximum	Length	Plastid genes carried
1	400,085	404,363	4,279	*ndhB* (partial)–*rps7*–*rps12–trnVGAC*
2	485,860	488,764	2,905	*trnPUGG–trnWCCA–petG–petL–psbE–psbF–psbL–psbJ (partial)*
3	745,633	747,636	2,004	*ycf2* (partial)*–trnICAU–rpl23-rpl2* (partial)
4	409,616	411,176	1,561	*psbD* (partial)–*psbC* (partial)
5	394,605	395,943	1,339	*ndhJ*–*ndhK* (partial)
6	659,032	661,065	1,245	*psbM*–*trnDGUC*–*trnYGUA*–*trnEUUC*
7	837,610	838,785	1,176	*infA* (partial)–*rps8*–*rpl14*
8	757,400	758,558	1,159	*trnAUGC*–*trnIGAU*
9	657,536	658,679	1,144	*petN*
10	543,561	544,412	852	*rrn16* (partial)
11	767,513	768,361	849	*rrn16* (partial)
12	754,944	755,663	720	*rpl2* (partial)
13	533,955	534,619	665	*trnIGAU* (partial)
14	96,326	96,851	526	*rrn23* (partial)
15	879,912	880,391	480	*ndhB* (partial)
16	262,147	262,584	438	*atpA* (partial)
17	949,466	949,903	438	*atpA* (partial)
18	96,995	97,431	437	*rrn23* (partial)
19	101,192	101,562	371	*rrn23* (partial)
20	544,905	545,182	278	*rrn16* (partial)
21	269,458	269,702	245	*clpP* (partial)
22	98,321	98,528	208	*rrn23* (partial)
23	190,158	190,337	180	*ycf2* (partial)
24	752,648	752,805	158	*rrn23* (partial)
25	872,552	872,694	143	*rrn23* (partial)
26	14,492	14,627	136	*rrn16* (partial)
27	29,848	29,980	133	*ycf2* (partial)
28	221,881	221,966	86	*trnNGUU*
29	71,527	71,612	86	*rrn23* (partial)
30	205,622	205,703	82	*trnHGUG*
31	98,988	99,067	80	*rrn23* (partial)
32	366,960	367,036	77	*trnICAU*
33	110,706	110,781	76	*trnMCAU*
34	885,128	885,203	76	*rrn23* (partial)
35	738,972	739,045	74	*trnfMCAU*
36	99,634	99,707	74	*rrn23* (partial)
37	739,435	739,507	73	*trnMCAU*
38	577,233	577,303	71	None
39	465,334	465,404	71	*trnfMCAU* (partial)
40	27,182	27,252	71	*trnfMCAU* (partial)
41	818,833	818,898	66	*atpE* (partial)
42	686,509	686,572	64	*trnPUGG* (partial)
43	213,524	213,587	64	*trnPUGG* (partial)
44	329,994	330,056	63	*rrn16* (partial)
45	31,362	31,422	61	*rrn16* (partial)
46	950,996	951,056	61	*rrn16* (partial)
47	260,996	261,056	61	*rrn16* (partial)
48	163,196	163,255	60	*rrn16* (partial)
49	583,586	583,641	56	*ndhH* (partial)
50	917,652	917,706	55	*trnFGAA* (partial)
51	675,036	675,088	53	*ndhI* (partial)
52	202,238	202,290	53	*atpB* (partial)
53	473,683	473,735	53	*trnQUUG* (partial)
54	545,277	545,327	51	*rrn16* (partial)

### Genome structure and conserved gene clusters

Vascular plant mt genomes are featured by structural dynamics with 31 rearrangements needed to reconcile the gene order of any two mt genomes [8]. The comparison of the mt gene orders of the five early angiosperms (S2 Table) in UniMoG [68] indicates that the gene order of *Magnolia* mt genome requires 31, 34, 34, and 44 rearrangements to get collinearity with that of the *Liriodendron*, *Schisandra*, *Amborella*, and *Nymphaea*, respectively. The repeat number in each mt genome and the divergence time of the three species related to *Magnolia* appear to be correlated with the number of rearrangement events [8, 65]. Despite structural fluidity, we observed several conserved gene clusters (e.g., *rpl2*–*rps19*–*rps3*–*rpl16*, *atp8*–*cox3*–*sdh4*, *nad3*–*rps12*, *rpl5*–*rps14*–*cob*, *rps13*–*nad1*.*x2*.*x3*, *trnSGCU*–*trnFGAA*–*trnPUUG*, *trnYGUA*–*nad2*.*x3*.*x4*.*x5*, <*nad5*.*x4*.*x5*><*trnETTC*–*nad7*>) in *Magnolia* mt genome compared with the gene order of that of the other angiosperms [69]. The retention of these gene orders across angiosperms [27, 30] despite the fast structural evolution over hundreds of millions of years might suggest certain selection forces and constraints upon the retention of these conserved gene clusters.

### Phylogenetic inference

The NT dataset is comprised of 38 protein-coding genes, adding up to 30,903 bp (missing data, ~9.8%), with 9,541 parsimony-informative sites (30.9%), which corresponds to 10,301 characters with 4,153 parsimony-informative sites in AA dataset. Partitionfinder recognized 15 and 9 subsets for NT and AA datasets, respectively.

Our phylogenetic reconstruction based on the NT dataset is largely congruent with the phylogeny of angiosperms reconstructed from four mitochondrial genes [70]. The corresponding AA dataset produced otherwise a novel topology (S4 Fig) with a paraphyletic magnoliids, and a polyphyletic Austrobaileyales, which might be explained by the amino-acid level homoplasy induced by strong selection for high hydrophobicity of the mitochondrial amino acids [70]. Therefore, nucleotide datasets might better reflect the organismal phylogeny in mitochondrial phylogenomic studies. The NT dataset generally produced better BS and PP support than AA in most of the nodes. Our analyses recovered strong BS and PP support for the majority of nodes in the current sampling scope. In all of our analyses, serial divergences of ANA grade (Amborelllales, Nymphaeales, Austrobaileyales) occurred at the base of angiosperm phylogeny, before the diversification of mesangiosperms. With the exception of *Ceratophyllum* that is not sampled in our study due to insufficient high-quality reads available in NCBI SRA database, the relationships among the four mesangiosperm clades (Chloranthales, magnoliids, eudicots, and monocots) sample here have weak to moderate BS support.

**Fig 2:**
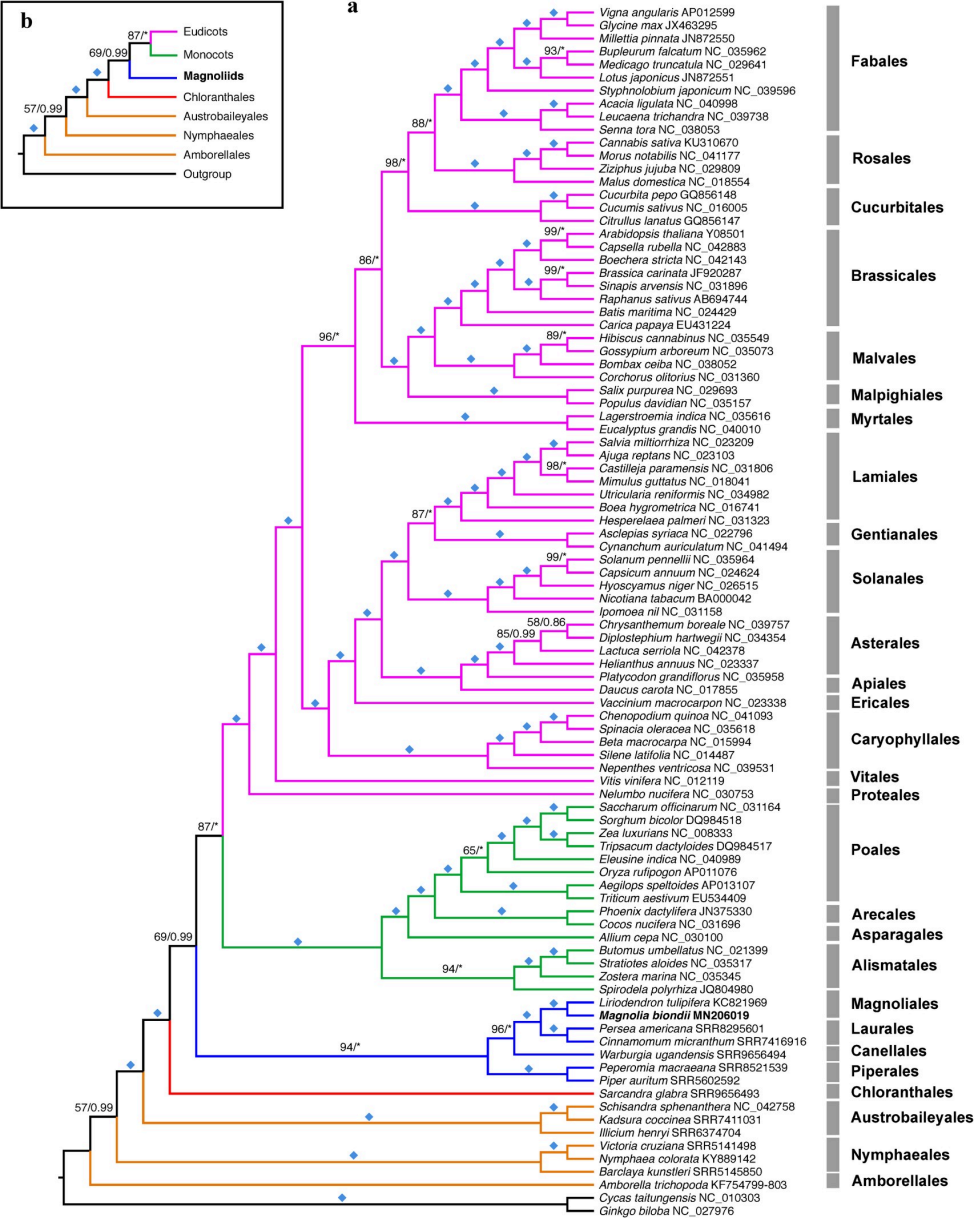
Phylogenetic tree inferred from the concatenated nucleotide dataset (NT). Asterisks indicate either BS of 100% or PP of 1.00. Diamonds indicate both BS of 100% and PP of 1.00. a) A detailed phylogeny of 93 taxa. Newly sequenced *Magnolia biondii* is highlighted in bold. b) An abbreviated tree showing the relationships of major lineages of early angiosperms. Branches representing eudicots, monocots, magnoliids, Chloranthales, and ANA grade are indicated in magenta, green, blue, red, and orange, respectively. Those branches with both BS and PP support below 50% were collapsed.

In NT dataset analyses (Fig 2), both monocots and eudicots receive 100% BS and 1.00 PP support. The sister relationship of monocots and eudicots has 87% BS and 1.00 PP support. All magnoliid taxa form a monophyletic group with 94% BS and 1.00 PP support, which is consistent with previous studies [70], albeit with stronger supports in our study. Within magnoliids, Canellales is strongly resolved as the sister to a clade containing Magnoliales and Laurales, rather than the sister to Piperales as in previous analyses [70, 71]. However, extended samplings might be needed to resolve the interordinal relationships within magnoliids. In contrast to the robust sister relationship of magnoliids with eudicots based on nuclear evidence [32, 34, 35], our study recovered a moderately-supported sister relationship of magnoliids with monocots and eudicots with 69% BS and 0.99 PP support in the nucleotide data analyses, which is also congruent with the plastid evidence [36, 37]. Therefore, organellar phylogenomic analyses tend to support the sister relationship of magnoliids with eudicots and monocots.

Our study shows that the mitochondrial phylogenomics are informative tools for resolving relationships among families, orders, or higher taxonomic ranks across angiosperms, especially for reconstruction of ancient phylogenetic relationships. However, some deep nodes, such as the phylogenetic divergence order of *Nymphaeales* and *Amborellales*, the relationship among the five mesangiosperm lineages, were not well resolved in the current analysis. Extended samplings of more representatives of the early angiosperms and the comparison of mt phylogeny with those of the plastid [36, 37], nuclear [34, 72], morphology, and non-molecular data would be essential to confidently revolve the phylogenetic relationships of magnoliids relative to monocots and eudicots.

## Conclusions

We assembled the draft mt genome of *Magnolia* using the Oxford Nanopore sequencing technology. The gene and intron content of *Magnolia* mt genome is similar to that of the *Nymphaea* and *Liriodendron* mt genomes, with *Magnolia* standing out by a relatively larger genome size packed with abundant repeated sequences, ancestrally retained sequences, and nuclear homologous sequences in its intergenic spacers. Despite high proportions of medium and large sized repeats, recombination activity is rather inert with only 17 recombinationally active repeats in the *Magnolia* mitochondrion. Repeat recombinations in the *Magnolia* mitochondrion could result in mtDNA heteroplasmy, hence contributing to dynamic structural evolution. Despite that, the *Magnolia* mt genome retains similar conserved gene clusters as *Liriodendron*, *Nymphaea*, *Schisandra*, and *Amborella*, suggesting unrecognized selection constraints on the retention of these gene clusters. This study allows new insight on the diversity and evolution of mitochondrial genomes in early flowering plants and repeat-mediated recombination patterns in plant mt genomes. Our study also provides mitochondrial evidences for the sister relationship of magnoliids with a clade comprising eudicots and monocots.

## Supporting information

S1 Table Sequencing statistics. (PDF)

S2 Table Comparison of mt gene content and gene order of the five early angiosperms. '>'s indicate lines of organism names. Chromosomes are circular if ended with ')', otherwise they are linear if without ')'s. Genes (name) start with '-'. indicate minus strand encoded genes, otherwise positive strand encoded genes.
(PDF)

S1 Fig The schematic illustrations of the read coverage of the *Magnolia biondii* mitochondrial genome of the (a) original linear mitochondrial genome contig; and (b) the putatively circular mitochondrial genome. The reads mapping files in bam format is visualized in Geneious and exported as the image files shown above.
(PDF)

S2 Fig The line plot and the genome map of the circular molecule of the putative mitochondrial genome of *Magnolia biondii*: (a) the line plot of the generation of the circular molecule; and (b) the genome map of the putative circular mitochodrial genome generated by OGDraw V1.2. Genes outside of the circle are transcribed clockwise, whereas those inside are transcribed counter-clockwise. Genes from the same protein complex are colored the same, introns are indicated in white boxes.
(PDF)

S3 Fig The flow chart for repeat recombination analysis of the repeated sequences in the mitochondrial genome of *Magnolia biondii*. (PDF)

S4 Fig Phylogenetic tree inferred from the amino acid (AA) dataset. Asterisks indicate either BS of 100% or PP of 1.00. Diamonds indicate both BS of 100% and PP of 1.00. a) A detailed phylogeny of 93 taxa. Newly sequenced *Magnolia biondii* is highlighted in bold. b) An abbreviated tree showing the relationships of major lineages of early angiosperms. Eudicots, monocots, magnoliids, Chloranthales, and ANA grade are marked in magenta, green, blue, red, and orange, respectively. Branches with both BS and PP support below 50% were collapsed.
(PDF)
