# Supplementary material for: The draft mitochondrial genome of Magnolia biondii and mitochondrial phylogenomics of angiosperms
Source: PLoS One. 2020 Apr 15;15(4):e0231020. doi: 10.1371/journal.pone.0231020 (PMC7159230; doi:10.1371/journal.pone.0231020)
Supplement: S1 Table — (PDF) [file pone.0231020.s001.pdf]

S1 Table. Sequencing statistics.

Sequencing	Library type	Raw data				Clean data			
		Read length (mean; max)	Total number of reads	Total number of bases (Gb)	Depth (X)	Read length (bp)	Total number of reads (bp)	Total number of bases (Gb)	Depth (X)
10 x Genomics	Genomic DNA	150	1,169,695,614	175.45	80.00	150	908,810,764	136.32	61.96
Oxford Nanopore	Genomic DNA	13,492; 1,123,682	12,836,970	174.10	79.00	14,839	5,858,689	86.94	39.52
illumina Hiseq 2000	RNA-seq Leaf	100	51,326,726	4.62	NA	100	25,703,370	2.20	NA
