# Supplementary material for: The draft mitochondrial genome of Magnolia biondii and mitochondrial phylogenomics of angiosperms
Source: PLoS One. 2020 Apr 15;15(4):e0231020. doi: 10.1371/journal.pone.0231020 (PMC7159230; doi:10.1371/journal.pone.0231020)
Supplement: S2 Table — '>'s indicate lines of organism names. Chromosomes are circular if ended with ')', otherwise they are linear if without ')'s. Genes (name) start with '-'. indicate minus strand encoded genes, otherwise positive strand encoded genes. (PDF) [file pone.0231020.s002.pdf]

S2 Table. Comparison of mt gene content and gene order of the five early angiosperms. '>'s indicate lines of organism names. Chromosomes are circular if ended with ')', otherwise they are linear if without ')'s. Genes (name) start with '-'. indicate minus strand encoded genes, otherwise positive strand encoded genes.

>*Liriodendron tulipifera*

rps10 atp8 cox3 sdh4 trnNGUU-cp atp1 rps7 rps13 nad1 -nad4 rps1 trnMCAU-cp nad4L atp4 trnQUUG trnDGUC-cp -trnICAU -nad2 atp9 trnSGCU trnFGAA trnPUGG sdh3 -nad1 -ccmB - nad6 -rrn26 -trnGGCC atp6 cox2 nad6 -nad5 trnEUUC nad7 -trnSUGA trnPUGG-cp trnWCCA-cp -nad5 - nad1 -matR -nad1 cox1 -ccmFN -rps2 trnMfCAU nad9 -trnVUAC -rps11 -trnKUUU -ccmFC -cob -rps14 - rpl5 -trnICAU nad5 nad3 rps12 trnCGCA trnHGUG-cp -tatC -trnPUGG -nad2 -trnYGUA rrn18 rrn5 -rpl16 - rps3 -rps19 -rpl2 ccmC rps4 rpl10)

>*Magnolia biondii*

rps10 atp6 -nad7 -trnETTC nad5 -nad6 -cox2 rpl2 rps19 rps3 rpl16 cox1 -ccmFN -rrn5 nad5 nad3 rps12 -sdh3 trnDGTC -nad5 -nad1 -matR -nad1 atp9 -rrn18 - ccmFC -trnWCCA -trnPTGG trnQTTG nad4 -nad1 -ccmB trnKTTT rps11 trnVTAC rpl5 rps14 cob rpl10 ccmC rps4 -nad9 -trnfMCAT -nad2 trnSTGA atp8 cox3 sdh4 trnNGTT trnYGTA nad2 tatC -trnHGTG - trnCGCA trnICAT -rps2 -nad1 -rps13 -trnMCAT -rps1 trnGGCC rrn26 rps7 atp1 nad4L atp4 -trnPTGG - trnFGAA -trnSGCT

>*Amborella trichopoda*

atp6 -atp8 -rps12 -nad3 nad7 nad1 -nad4L atp4 -rps11 -rpl16 - rps3 -rps19 -rpl2 -atp1 trnNGUU trnPUGG sdh3 nad4 nad6 cox2 rrn26 trnfMCAU -nad2 -rps7 atp9 ccmFN rpl5 rps14 cob nad5 -mttB nad9 -trnKUUU rps2 -nad5 nad5 rps4 ccmC -rps1 trnDGUC trnHGUG —nad2 - nad1 -matR -trnSUGA -trnWCCA trnMCAU rrn18 rrn5 nad1 ccmFC cox3 sdh4) rps10 cox1 rps13 - trnPUGG -nad1)

>*Nymphaea colorata*

-atp9 -rps7 rps4 -rpl10 -rps19 -nad5 trnEUUC nad7 nad4L nad6 - cox1 -rps10 rpl5 rps14 cob -trnMCAU -trnNGUU-cp -atp6 -nad1 -trnMCAU -trnYGUA -trnKUUU -atp1 - trnCGCA -trnDGUC -mttB ccmC cox3 sdh4 ccmFC -atp4 -cox2 ccmFN -nad9 -trnLCAA matR nad1 trnPUGG trnWCCA -atp8 -nad4 nad2 atp6 -trnFGAA -trnGGCC -trnfMCAU -rrn26 —trnHGUG -sdh3 — trnPUGG -rrn5 -rrn18 -nad1 -rps2 -nad1 trnQUUG -rpl16 -rps3 -rps19 -rpl2 trnWCCA nad3 rps12 nad5 - ccmB -rps13 -rps11 -trnSUGA rps1 nad2 -trnFGAA)

>*Schisandra sphenanthera*

trnCGCA rps7 -trnHGUG trnPUGG rps2 atp6 atp8 rps4 rrn18 rrn5 -trnGGCC sdh4 -rps11 -trnEUUC -nad2 trnHGUG atp4 trnEUUC nad7 ccmFN nad2 trnfMCAU trnNGUU nad4L atp9 trnMCAU nad1 -nad5 -ccmC -atp9 -nad1 trnDGUC nad1 trnPUGG rps10 cox1 -nad4 rps13 -cox2 -atp6 matR nad1 nad5 nad3 rps12 cox3 -ccmB trnKUUU trnWCCA -rrn26 trnfMCAU rpl2 rps3 rpl16 trnfMCAU ccmFC trnSGCU trnFGAA trnPUGG sdh3 -cob - rps14 -rpl5 -rps1 -mttB rpl10 -trnFGAA -trnSGCU -trnYGUA)
