# Supplementary material for: The draft mitochondrial genome of Magnolia biondii and mitochondrial phylogenomics of angiosperms
Source: PLoS One. 2020 Apr 15;15(4):e0231020. doi: 10.1371/journal.pone.0231020 (PMC7159230; doi:10.1371/journal.pone.0231020)
Supplement: S1 Fig — The reads mapping files in bam format is visualized in Geneious and exported as the image files shown above. (PDF) [file pone.0231020.s003.pdf]

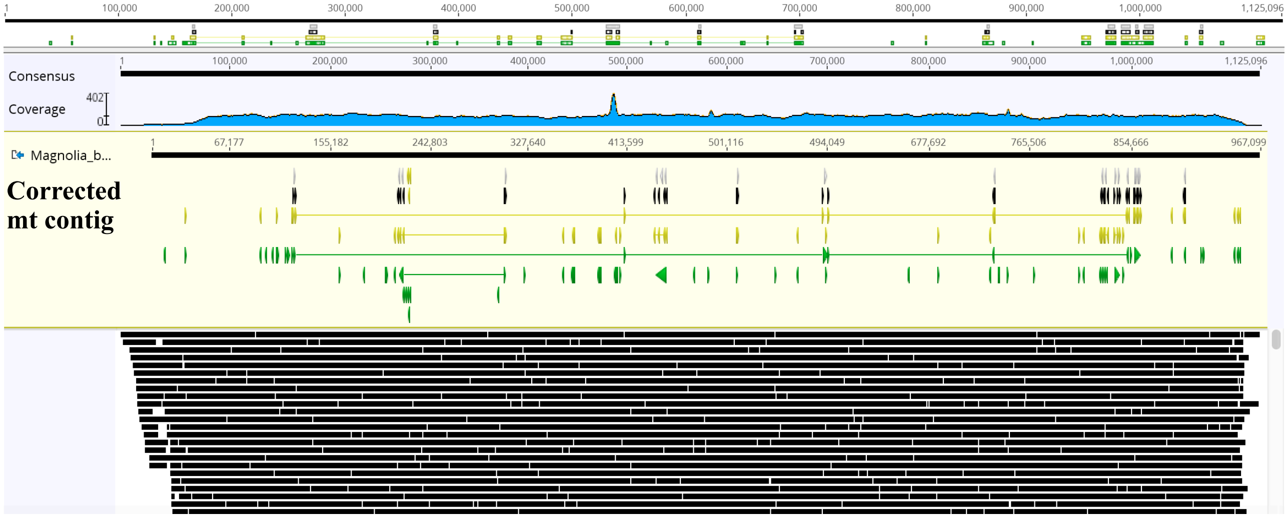

(a)

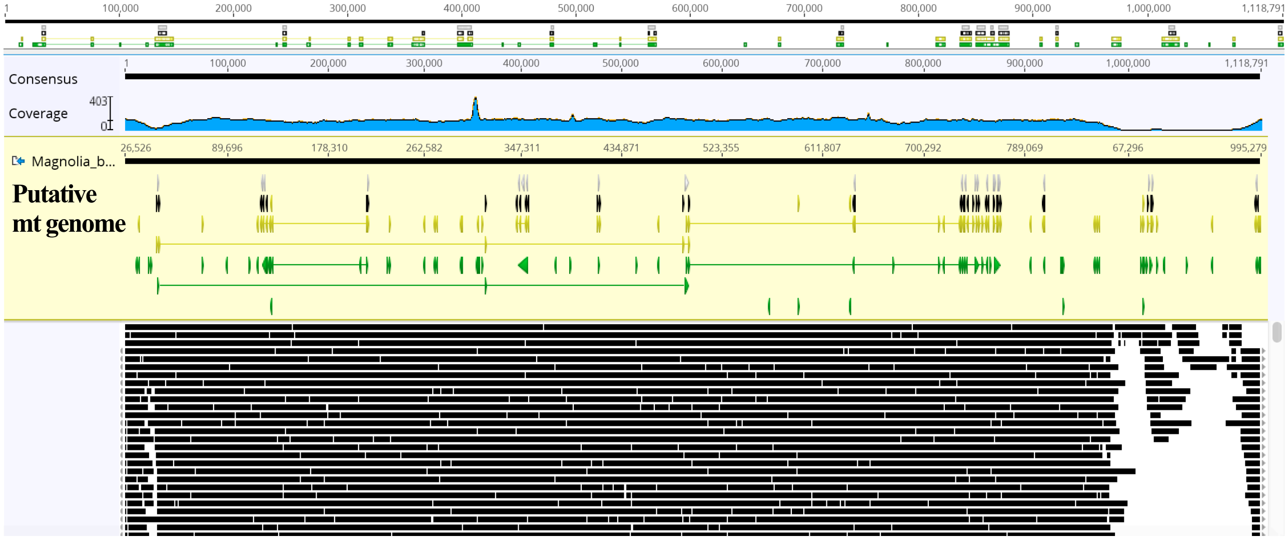

(b)

S1 Fig. The schematic illustrations of the read coverage of the *Magnolia biondii* mitochondrial genome of the (a) original linear mitochondrial genome contig; and (b) the putatively circular mitochondrial genome. The reads mapping files in bam format is visualized in Geneious and exported as the image files shown above.
