# Supplementary material for: The draft mitochondrial genome of Magnolia biondii and mitochondrial phylogenomics of angiosperms
Source: PLoS One. 2020 Apr 15;15(4):e0231020. doi: 10.1371/journal.pone.0231020 (PMC7159230; doi:10.1371/journal.pone.0231020)
Supplement: S2 Fig — The line plot and the genome map of the circular molecule of the putative mitochondrial genome of Magnolia biondii: (a) the line plot of the generation of the circular molecule; and (b) the genome map of the putative circular mitochodrial genome generated by OGDraw V1.2. Genes outside of the circle are transcribed clockwise, whereas those inside are transcribed counter-clockwise. Genes from the same protein complex are colored the same, introns are indicated in white boxes. (PDF) [file pone.0231020.s004.pdf]

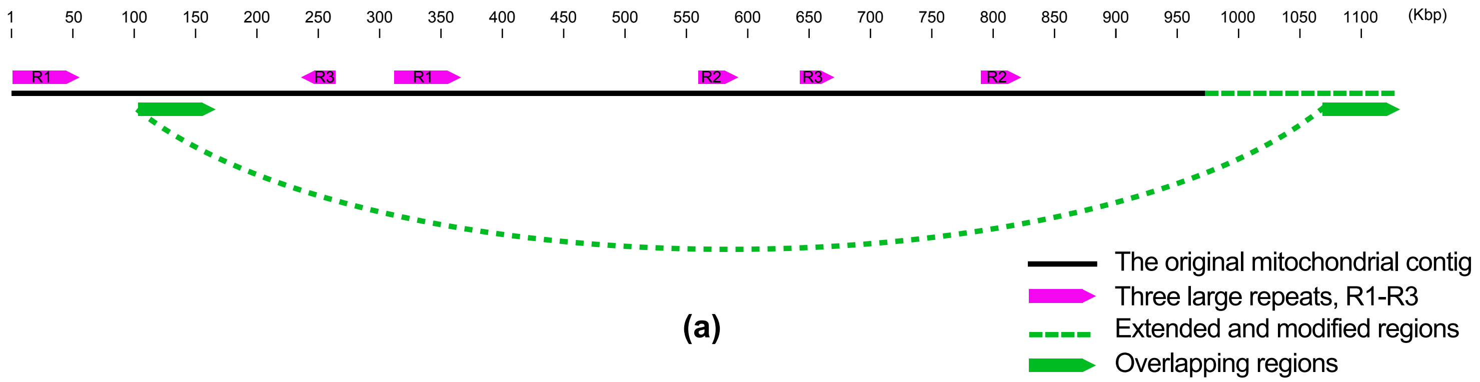

(a)

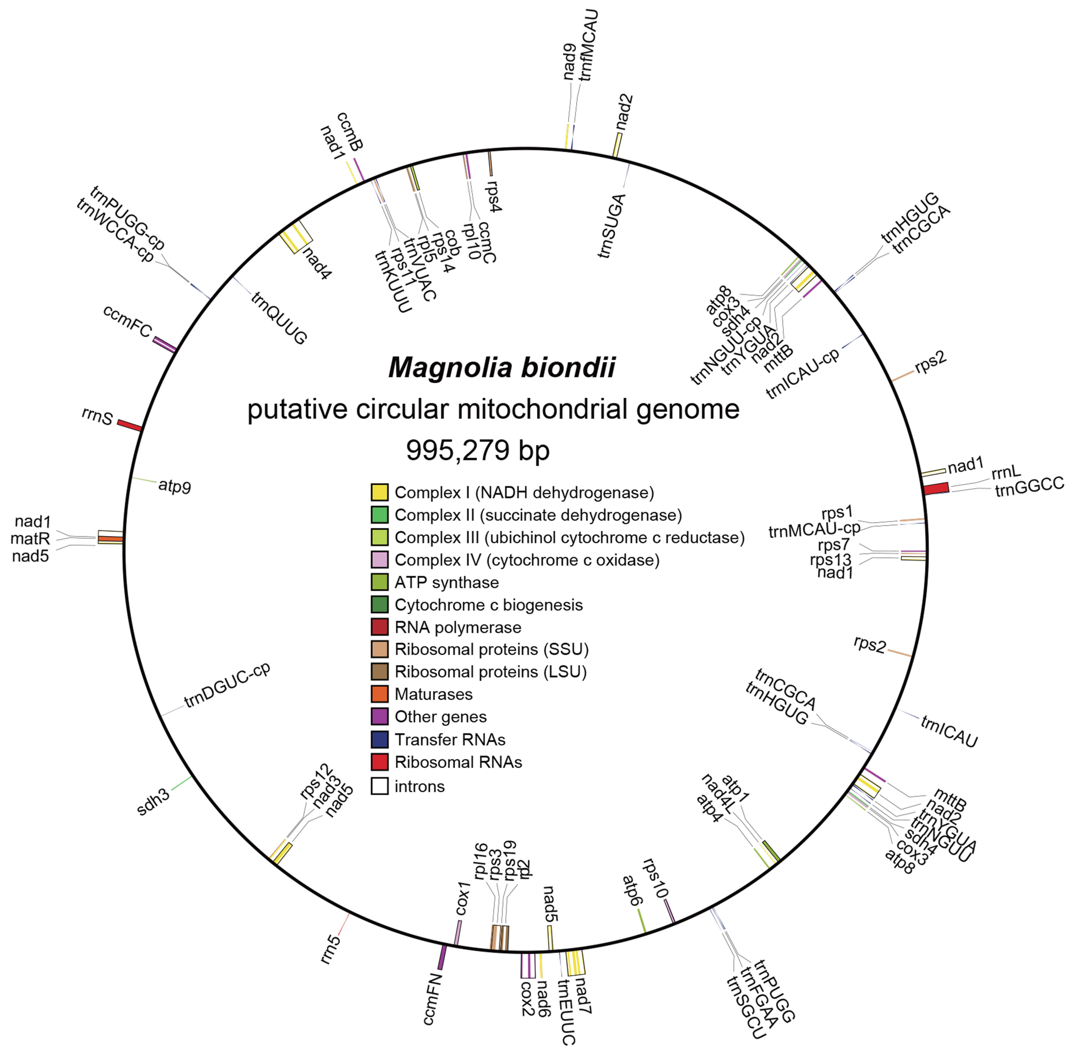

(b)

S2 Fig. The line plot and the genome map of the circular molecule of the putative mitochondrial genome of *Magnolia biondii*: (a) the line plot of the generation of the circular molecule; and (b) the genome map of the putative circular mitochodrial genome generated by OGDraw V1.2. Genes outside of the circle are transcribed clockwise, whereas those inside are transcribed counter-clockwise. Genes from the same protein complex are colored the same, introns are indicated in white boxes.
