# Supplementary material for: The draft mitochondrial genome of Magnolia biondii and mitochondrial phylogenomics of angiosperms
Source: PLoS One. 2020 Apr 15;15(4):e0231020. doi: 10.1371/journal.pone.0231020 (PMC7159230; doi:10.1371/journal.pone.0231020)
Supplement: S3 Fig — (PDF) [file pone.0231020.s005.pdf]

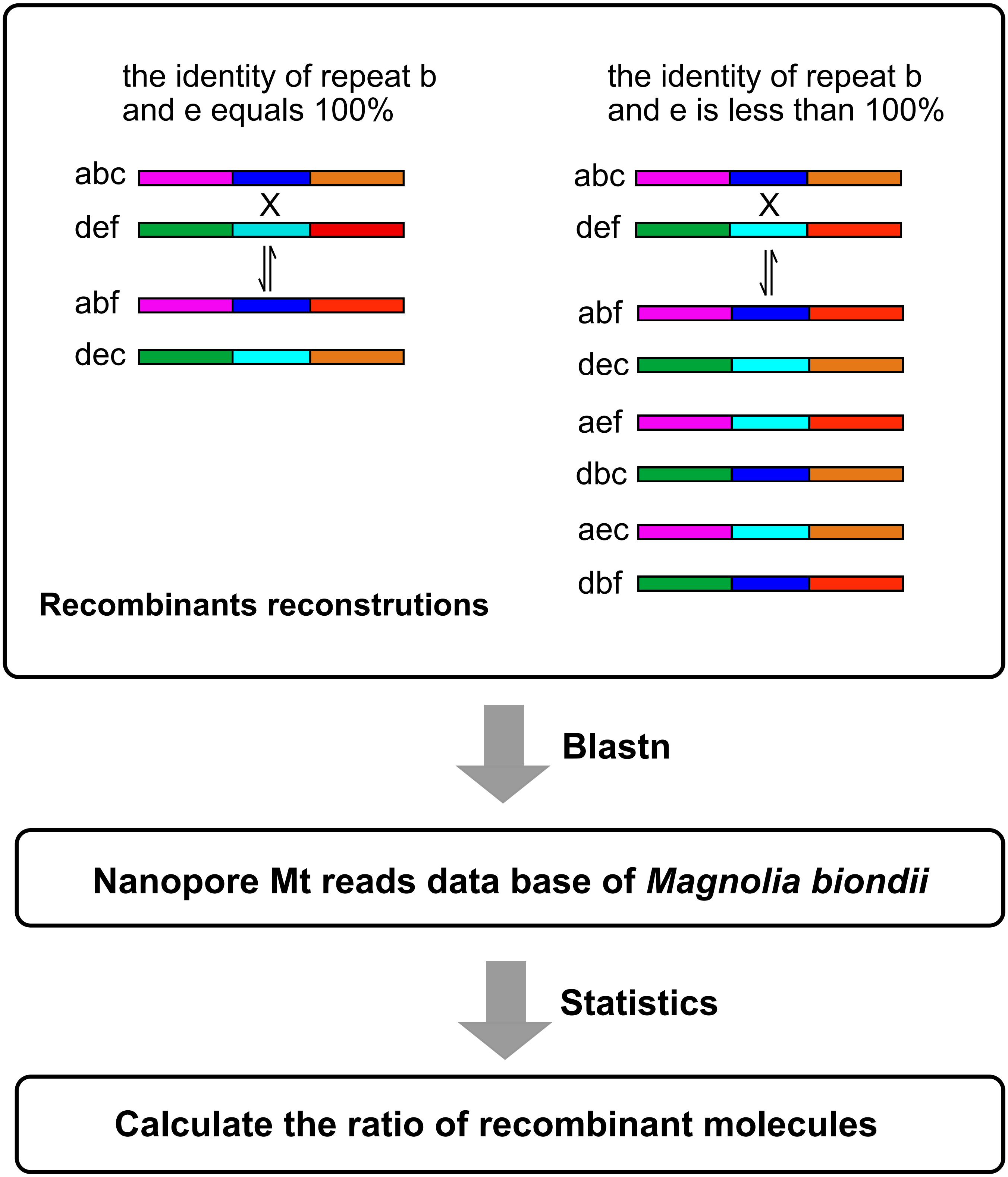

S3 Fig. The flow chart for repeat recombination analysis of the repeated sequences in the mitochondrial genome of *Magnolia biondii*.
