# Supplementary material for: The draft mitochondrial genome of Magnolia biondii and mitochondrial phylogenomics of angiosperms
Source: PLoS One. 2020 Apr 15;15(4):e0231020. doi: 10.1371/journal.pone.0231020 (PMC7159230; doi:10.1371/journal.pone.0231020)
Supplement: S4 Fig — Asterisks indicate either BS of 100% or PP of 1.00. Diamonds indicate both BS of 100% and PP of 1.00. a) A detailed phylogeny of 93 taxa. Newly sequenced Magnolia biondii is highlighted in bold. b) An abbreviated tree showing the relationships of major lineages of early angiosperms. Eudicots, monocots, magnoliids, Chloranthales, and ANA grade are marked in magenta, green, blue, red, and orange, respectively. Branches with both BS and PP support below 50% were collapsed. (PDF) [file pone.0231020.s006.pdf]

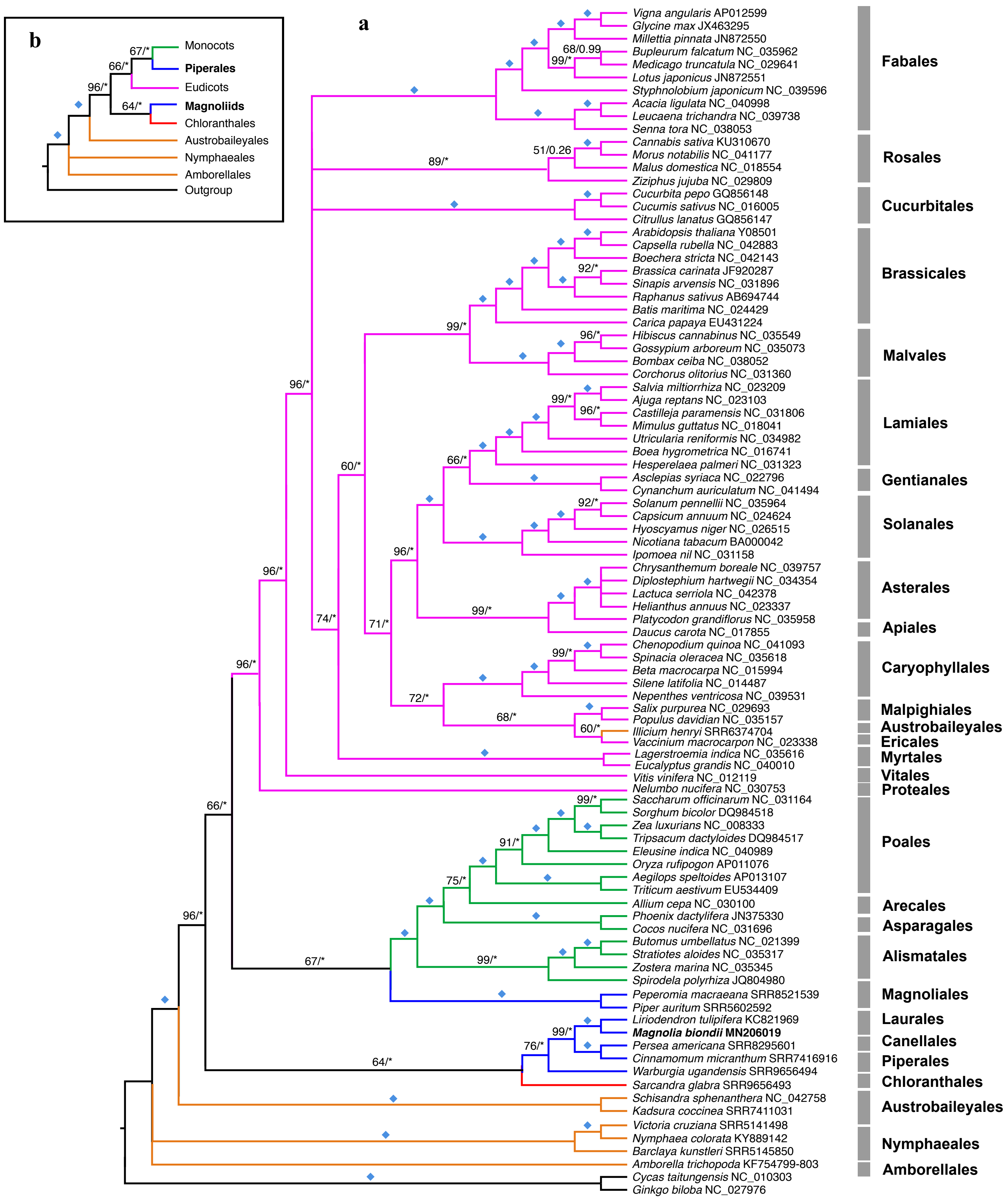

S4 Fig. **Phylogenetic tree inferred from the amino acid (AA) dataset**. Asterisks indicate either BS of 100% or PP of 1.00. Diamonds indicate both BS of 100% and PP of 1.00. a) A detailed phylogeny of 93 taxa. Newly sequenced *Magnolia biondii* is highlighted in bold. b) An abbreviated tree showing the relationships of major lineages of early angiosperms. Eudicots, monocots, magnoliids, chloranthales, and ANA grade are marked in magenta, green, blue, red, and orange, respectively. Branches with both BS and PP support below 50% were collapsed.
